# Water impacting on superhydrophobic macrotextures

**DOI:** 10.1038/ncomms9001

**Published:** 2015-08-11

**Authors:** Anaïs Gauthier, Sean Symon, Christophe Clanet, David Quéré

**Affiliations:** 1 Physique and Mécanique des Milieux Hétérogènes, UMR 7636 du CNRS, ESPCI, 75005 Paris, France; 2 LadHyX, UMR 7646 du CNRS, École polytechnique, 91128 Palaiseau, France

## Abstract

It has been recently shown that the presence of macrotextures on superhydrophobic materials can markedly modify the dynamics of water impacting them, and in particular significantly reduce the contact time of bouncing drops, compared with what is observed on a flat surface. This finding constitutes a significant step in the maximization of water repellency, since it enables to minimize even further the contact between solid and liquid. It also opens a new axis of research on the design of super-structures to induce specific functions such as anti-freezing, liquid fragmentation and/or recomposition, guiding, trapping and so on. Here we show that the contact time of drops bouncing on a repellent macrotexture takes discrete values when varying the impact speed. This allows us to propose a quantitative analysis of the reduction of contact time and thus to understand how and why macrotextures can control the dynamical properties of bouncing drops.

## Abstract (web summary)

The behaviour of water impacting on surfaces is crucial to many applications, including anti-icings and self-cleaning. Here, Gauthier *et al.* show that the contact time of drops on a straight wire decreases with impact speed in a step manner due to the fragmentation of water in evenly divided subunits.

## Graphical abstract

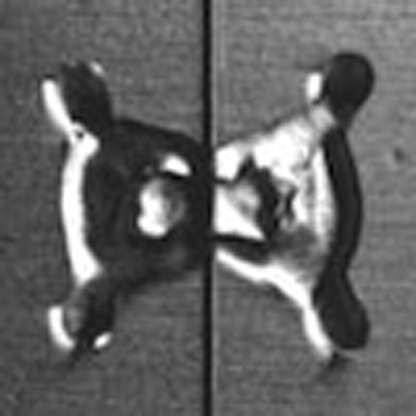

A hydrophobic material textured at a microscopic scale exhibits spectacular dynamical properties, to which correspond different functionalities used in various natural systems^1^. Water is observed to bounce after an impact^2^,^3^,^4^, which can lead to anti-icing properties if takeoff occurs before freezing^3^,^5^,^6^. At the small scale of dew, hydrophobic nanotextured surfaces can expel micrometric droplets as they coalesce with their neighbours, demonstrating anti-fogging abilities^7^,^8^. Water is observed to roll and glide on water-repellent materials at velocities typically 100 times larger than on usual materials^9^, a high mobility that induces self-cleaning, as shown on lotus leaves and for aphids^1^,^10^,^11^.

Contrasting with the quest of original properties obtained by reducing the texture size^7^,^8^, it was recently reported that macrotextures (in the range of 100 μm to 1 mm) also induce novel behaviours. First, examples of this kind were proposed in the Leidenfrost state, where superhydrophobicity arises from heating the substrate. Millimetric crenellations are observed to enhance friction, allowing one to control the levitating liquid^12^. More remarkably, making crenels asymmetric causes the self-propulsion of volatile liquids placed upon such hot ratchets^13^, an effect that attracted a lot of attention since its discovery^14^,^15^,^16^,^17^. New behaviours were also evidenced at room temperature on superhydrophobic materials decorated with macrotextures. Grooves of submillimetric width and depth etched at the surface can guide liquid moving at the surface^18^, and water projected against the edge of a blade can be split^19^ or cut when the blade is used as a knife^20^.

Impact is also affected by the presence of large defects: sub-millimetre ridges^3^, big conical posts^4^ or large chemical defects^21^ on a water-repellent material markedly modify the bouncing dynamics by reshaping the liquid at impact and takeoff, which can reduce by a large amount the contact time of rebounding droplets^3^,^4^. We focus here on such rebounds and consider the impact of water drops on superhydrophobic wires placed along flat materials of same repellency. Bird *et al.* recently showed that ridges can divide by a factor of order 2 the contact time *τ* of bouncing drops^3^, and we investigate how this reduction varies with impact velocity *V*, drop size and macrotexture radius, which leads to a scenario for understanding the effect. We show that the contact time in presence of the macrotexture decreases with *V* in a step-like fashion, exhibiting two reduced values at high and intermediate velocity. Using top and side views of the bouncing, we interpret this result by the formation of 4, 2 or 1 main liquid subunits during impact, so that bouncing time *τ* is divided by 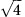, 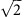 or 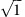, compared with that on regular repellent surfaces. Extensions to other designs of the macrotexture are finally shown to provide other shapes at impact, and thus other contact times.

## Results

### Bouncing dynamics

**Figure 1:**
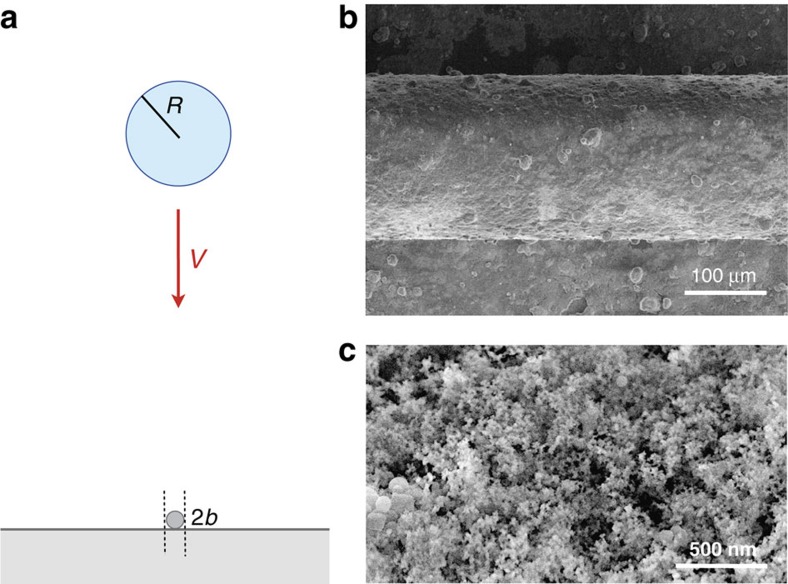
Experimental set-up and materials. (**a**) A water drop of radius *R* (between 1 and 2.3 mm) impacts at velocity *V* (between 20 and 150 cm s^−1^) a wire of radius *b*<*R* held along a flat surface. Both the substrate and the wire are treated with the same hydrophobic colloidal suspension, making them similarly water repellent. (**b**) Scanning electron microscope image of the substrate with a wire of radius *b*=100 μm. (**c**) Close-up view of the texture, showing aggregates of colloidal beads of typical size 20 nm.

A nickel wire of radius *b* (between 25 and 250 μm) is placed horizontally along an aluminium substrate (Fig. 1a). The whole system is treated using a commercial spray (Ultra Ever Dry, UtraTech International) consisting of hydrophobic nanoparticles dispersed in acetone. After solvent evaporation, micrometric layers of nanoparticles coat the substrate and the wire (Fig. 1b,c), making both of them similarly superhydrophobic. Advancing and receding contact angles of water are found to be 166°±4° and 159°±1°, showing the conjunction of high angles and low hysteresis characteristic of a Cassie state^1^.

**Figure 2:**
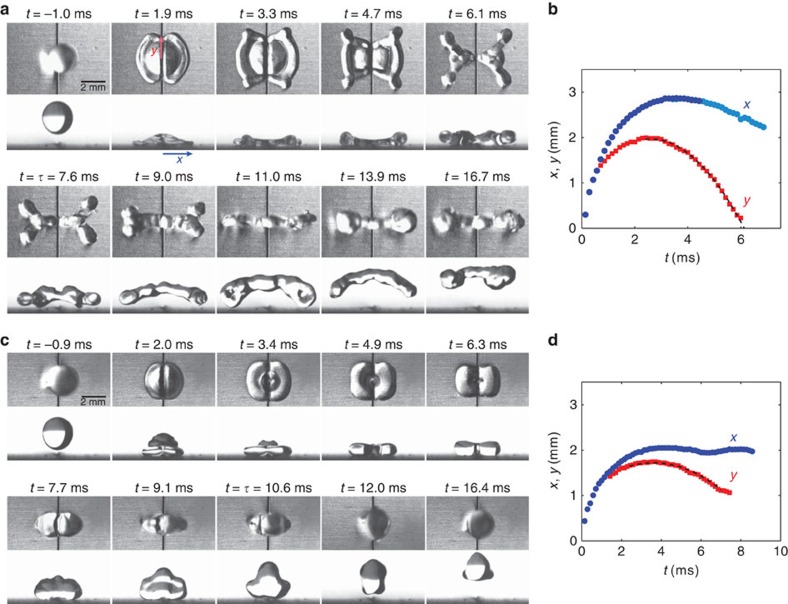
Dynamical behaviour of water drops bouncing on a repellent macrotexture. Top and side views of water drops (*R*=1.3 mm) impacting a superhydrophobic wire of radius *b*=100 μm maintained on a superhydrophobic surface. Corresponding movies are Supplementary Movies 1–4. (**a**) At an impact velocity *V*=1.0 m s^−1^, the drop adopts a butterfly shape before taking off at *t*=*τ*=7.6±0.2 ms, instead of *τ*_o_*=*13.0±0.8 ms without the wire. (**b**) Temporal variation of the positions *x* and *y* of drop's edges perpendicular and along the wire. Dark- and light-blue data are extracted from side and top views, respectively, and the dashed line is a parabolic fit. (**c**) At an impact velocity *V*=0.5 m s^−1^, the drop is much less deformed, and it leaves the substrate at *t*=*τ*=10.6±0.5 ms. (**d**) Distances *x* and *y* as a function of time. The dashed line is a parabolic fit.

Water drops of density *ρ*=1,000 kg m^−3^ and surface tension *γ*=72 × 10^−3^ N m^−1^ are formed from calibrated needles, allowing us to vary the radius *R* between 1 and 2.3 mm. For each needle, *R* is repeatable with <10% error. Impact velocity *V* is varied between 20 and 150 cm s^−1^ by adjusting the height from which drops are released. Impacts are filmed from above and from the side using a high-speed video camera (Phantom V9) at typically 10,000 fps. Figure 2 shows top and side views of water drops of radius *R*=1.3 mm impacting a substrate macrotextured by a straight wire of radius *b*=100 μm (the corresponding movies are Supplementary Movies 1–4). Impacts are always centred, and the presence of a wire makes the successive liquid shapes anisotropic. We also report in the figure the temporal variation of the positions *x* and *y* of the drop edges along and perpendicular to the wire, as defined in the second photo. Origin of time is chosen when the south pole of the drop contacts the wire.

In Fig. 2a, the impact velocity is 1.0 m s^−1^. A first striking observation is the very modest spreading along the wire, which quickly generates cusps at *y*≈*R* followed by a butterfly shape (*t*=3−5 ms). In this stage, the liquid impacting the wire bifurcates and gets expelled, so that four globules appear at the corners of the spreading film. Simultaneously, water starts to dewet the wire, as reported by Bird *et al.*^3^ who interpreted this early motion as a consequence of the film thinness above the macrotexture. As seen in Fig. 2b, axial dewetting captured by the curve *y*(*t*) occurs at a constant acceleration (parabolic dotted line), found to be *a=*160±10 m s^−2^. This behaviour contrasts with usual dewetting: here there is no growing rim since the liquid can escape by the wire sides, hence there is a constant acceleration instead of a constant velocity. Assuming a dewetting force 2*π*γ*b* along the wire, Newton's law *ρωa*=2*π*γ*b* provides a dewetting volume *ω*≈0.28 μl indeed comparable to 0.24±0.05 μl, the volume of each pair of arms linking cusp and lobe at *t*=3−5 ms in Fig. 2a.

Axial dewetting produces an outward flow, which, as it impacts the inward flow associated with the recoiling of the rest of the drop, makes water take off. Contact time *τ* is determined from side views when the last points of contact leave the substrate, which happens simultaneously for centred impacts. Takeoff in Fig. 2a occurs at *τ*=7.6±0.2 ms. Globules at this moment are still connected by a filament that breaks later, as water rises (Supplementary Movies 1 and 2). After takeoff, fragments seen from the side apparently grow (around *t*=11 ms), owing to the coalescence of lobes into more compact shapes, as observed from the top. Phenomena at larger speeds are similar, the only difference being that drops split during impact above *V*=1.1 m s^−1^ (for *R*=1.3 mm), which does not affect the value of *τ*.

For slower impacts, the sequence can be quite different as seen in Fig. 2a for *V* = 0.5 m s^−1^. Water spreads less and it adopts a square-like shape, between *t*=3.4 and 4.9 ms. Again, dewetting is faster along the wire (with again a constant acceleration found from the fit in Fig. 2d to be 51±3 m s^−2^), which thickens the two lobes lying on both sides of the wire and enables the rebound of these lobes, still connected by a bridge. The contact time *τ* is now found to be *τ*=10.6±0.5 ms, a value significantly larger than that of the experiment in Fig. 2a. After bouncing, the thick connection between the two globules allows them to merge, so that one single drop recomposes (Supplementary Movies 3 and 4).

### Contact time

**Figure 3:**
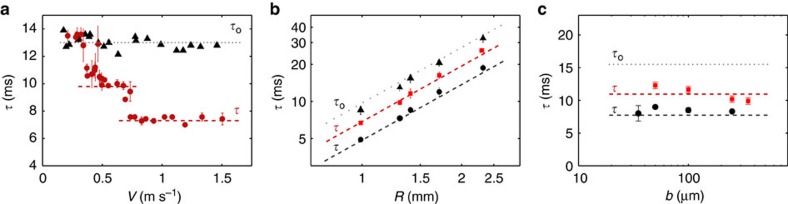
Contact time of bouncing drops with and without macrotexture. Contact time of water drops bouncing on a regular superhydrophobic material (time *τ*_o_), and on a similar surface with a wire of same repellency (time *τ*). (**a**) *τ*_o_ (black triangles) and *τ* (red circles) are plotted as a function of impact velocity *V*; dotted and dashed lines are fits for *τ*_o_ and *τ*. Drop radius *R* is 1.3 mm and wire radius *b* is 100 μm. Error bars come from non-simultaneous detachment of drops as they take off. (**b**) *τ*_o_ and *τ* are plotted as a function of the drop radius *R*, for *b*=100 μm. The reduced time *τ* is drawn at both intermediate *V* (*V*=0.5 m s^−1^, red squares) and high *V* (*V*=0.8 m s^−1^, black circles). Data are fitted by the equation *τ*_o_=2.6 (*ρR*^3^/*γ*)^1/2^ (dotted line), as found in ref. 2, and red and black dashed lines are *τ*=*τ*_o_/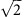 and *τ*=*τ*_o_/2, as proposed further in the paper. (**c**) Similar set of data, where the reduced time *τ* measured at intermediate (*V*=0.5 m s^−1^) and high (*V*=0.8 m s^−1^) velocity is plotted as a function of *b*, for a water drop of radius *R*=1.4 mm. Lines are defined as in **b**. In **b** and **c** the error bars represent the mean variations of *τ* in the plateaus seen in **a**.

We varied systematically the impact velocity *V* between 0.25 and 1.5 m s^−1^, and measured the contact time to determine how *τ* varies as a function of *V*. We performed similar experiments on a superhydrophobic material without macrotexture in the same interval of velocities and compared the corresponding contact time *τ*_o_ with the time *τ* in the presence of a wire. We plot in Fig. 3a *τ*_o_ and *τ* as a function of the impact velocity *V.* The reference contact time *τ*_o_ (black triangles) does not depend on *V*, in agreement with previous observations that suggested *τ*_o_ scales as the inertio-capillary time (*ρR*^3^/*γ*)^1/2^, where *ρ* and *γ* denote the liquid density and surface tension, respectively^2^. The precise value of *τ*_o_ (around 13 ms for *R*=1.3 mm) also agrees with other measurements for water drops of the same size^1^,^9^. In the presence of macrotexture (of radius *b*=100 μm), the law *τ*(*V*) is strikingly modified (red circles). At high impact velocity, the contact time also plateaus, yet at a much lower value than *τ*_o_. The time *τ*≈0.56 *τ*_o_ (dotted line) is typically reduced by a factor of 2, as reported by Bird *et al*^3^. for an impact velocity *V*=1.2 m s^−1^. This reduction is found here to persist in a wide range of impact velocities between 0.7 and 1.5 m s^−1^. However, another behaviour is observed when *V* is smaller than 0.7 m s^−1^. Below this value, the contact time critically increases and reaches another short plateau. The value of *τ* in this regime remains significantly smaller than *τ*_o_, by about 25%. Decreasing *V* even more leads to another step (around 0.4 m s^−1^), such that *τ* becomes comparable to *τ*_o_ (see Supplementary Movie 5). This step-like shape of the reduced contact time appears for each drop and wire radius we considered, as shown in Supplementary Fig. 1.

These observations are completed by measuring reduced contact times as a function of drop size *R* (Fig. 3b) and macrotexture radius *b* (Fig. 3c). We varied the drop size by a factor of 2.3: the drop must remain larger than the macrotexture, and smaller than the capillary length, so that this factor is close to the maximum possible case. We report in Fig. 3b contact times in both regimes of intermediate (*V*=0.5 m s^−1^, red squares) and high (*V*=0.8 m s^−1^, black circles) impact velocities. Data are also compared with linear fits in this log–log representation. Whatever the regime, *τ* varies as *R*^3/2^, the inertio-capillary scaling known for impact on flat substrates^2^,^3^,^4^, and was found here to resist the presence of a macrotexture. This scaling does not depend on *b*, and Fig. 3c shows that reduced contact times in the same regimes of impact velocity (and for *R*=1.4 mm) indeed hardly vary with the wire radius *b.* No variation is observed at high *V* (black circles), and *τ* decreases by about 25% when increasing *b* by a factor of 7 at intermediate *V* (red squares). Using a wire of radius *b*=25 μm in this regime brings us back to the contact time *τ*_o_ and to classical behaviours of rebound as if there were no texture: if the spreading film becomes thicker than the wire radius, we do not expect significant effects on the rebound.

## Discussion

How can we understand the reduction of contact time, and its step-like variation with impact velocity (Fig. 3a)? Following Bird *et al.*^3^, contact time can be expressed as the sum of a spreading time *τ*_s_ (until the liquid reaches its maximum size *R*_max_) plus a recoiling time *τ*_r_ scaling as *R*_max_/*U*, where *U* denotes an average retraction velocity. As seen in Fig. 2a, a liquid arch forms at the end of spreading, which signifies the existence of an air cavity along the wire. Since dewetting can start from both the wire and the edge, recoiling distance is divided by 2 compared with regular rebounds. Assuming a dewetting velocity similar with and without macrotexture and neglecting *τ*_s_ implies that *τ*=*τ*_s_+*τ*_r_≈*τ*_r_ is reduced by 50%. However, this argument is only qualitative: Fig. 2b shows that spreading can last roughly half the contact time, and, as pointed out by Bird *et al.*, recoiling stages are very different with a macrotexture pushing water away from it, causing dewetting dynamics to be quite different from those of usual films.

Despite its apparent complexity, the problem can be remarkably simplified owing to its inertio-capillary nature. On a regular superhydrophobic material, the contact time *τ*_o_ is independent of impact speed (Fig. 3a), although changing *V* induces a large variety of modes of deformation during contact. Regardless of the details of impact, inertia and capillarity are constantly antagonistic, so a drop of volume Ω behaves as a spring of mass *ρ*Ω and stiffness *γ*, which provides a contact time *τ*_o_ scaling as (*ρ*Ω/*γ*)^1/2^∼(*ρR*^3^/*γ*)^1/2^, as observed in Fig. 3b. When water hits a slender macrotexture at ‘large' velocity, it creates two half-films, which quickly reconfigure in four lobes (visible as soon as *t*=1.9 ms in Fig. 2a, and developing until *t*=*τ*). Due to its non-wetting character, the macrotexture initially deflects water and later expels it, which feeds the lobes. Hence, we have four subunits of liquid, each of them subjected to inertia and capillarity. With the volume of each subunit being Ω/4, we expect a contact time *τ*≈*τ*_o_/2, which is close to the behaviour observed experimentally in Fig. 3a where the fit (dashed line) provides *τ*≈7.3±0.2 ms while *τ*_o_/2 is 6.5±0.3 ms.

This geometrical argument allows us to understand the successive rises of contact time at lower impact velocity. We observe in Fig. 3a a first step around *V*=0.7 m s^−1^ that brings *τ* between *τ*_o_/2 and *τ*_o_. Water spreads less in this regime of intermediate velocity, and it remains more isotropic (Fig. 2c), without the cusps seen at high velocity (Fig. 2a). Hence, there are now just two subunits on each side of the wire. As a consequence, the inertio-capillary balance written on each subunit of volume Ω/2 suggests a contact time *τ*≈*τ*_o_/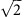. In Fig. 3a, *τ* in this regime is 9.8±0.3 ms (dashed line) indeed comparable to *τ*_o_/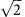
≈9.2±0.4 ms. At very low impact velocity (*V*<0.4 m s^−1^), the drop always remains thicker than the wire and the macrotexture no longer influences bouncing: water slightly deforms and recomposes without being divided, so we recover the bouncing time associated with a volume Ω, that is, *τ*_o_. This global approach also suggests that contact time in both regimes where it is reduced should obey the same scaling as *τ*_o_∼(*ρR*^3^/*γ*)^1/2^. This is indeed observed in Fig. 3b, where data are nicely fitted by such behaviours, as shown by red and black dashed lines, respectively, showing *τ*≈*τ*_o_/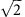 and *τ*≈*τ*_o_/2. The same lines drawn in Fig. 3c also fairly fit the data.

**Figure 4:**
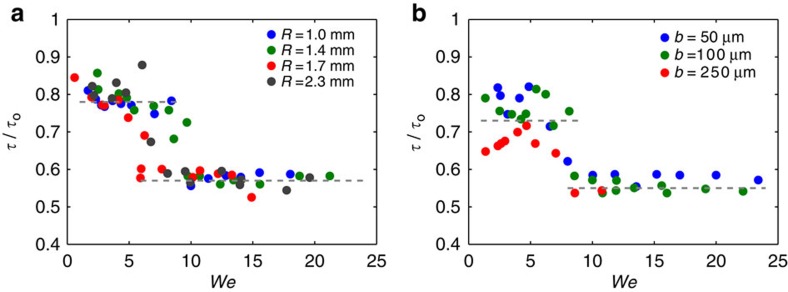
Step between the two plateaus of reduced contact time. Contact time *τ* on a macrotextured surface normalized by the contact time *τ*_0_ on a flat surface, as a function of the Weber number *We* in the intermediate- (two lobes) and high- (four lobes) velocity regimes. (**a**) Impact on a wire of radius *b*=100 μm for various drop radii *R* between 1.0 and 2.3 mm. (**b**) Impact of droplets of radius *R*=1.4 mm on wires of various radii between 50 and 250 μm. In both cases, data collapse on a common curve, showing a transition between the two regimes of reduced time at *We*=7.5±1.5.

Having determined the different contact times allows us to discuss the origin of the critical velocities between the regimes exhibited in Fig. 3a. The threshold at low *V* (transition one/two lobes) should occur when the impact velocity becomes large enough to split the drop, that is, when inertia overcomes capillary action. This corresponds to *We*≈1, where *We*=*ρV*^2^*R*/*γ* is the Weber number at impact. For a water drop of radius *R*=1.3 mm, this criterion yields *V*≈25 cm s^−1^, close to the observed value. At high *We*, the wire naturally generates four lobes, and the transition two/four lobes happens when the four lobes have enough time during contact to merge into two subunits. Lobes initially separated by a distance *αR*_max_ (*α*<1) are expected to move towards each other at a velocity *u* scaling as (*γ*/*ρR*)^1/2^. If the time *αR*_max_/*u* is smaller than *τ* (whose scaling has just been discussed), the lobes merge before bouncing in two bigger subunits, which defines the regime of intermediate contact time. Since *R*_max_ itself is of the form *R* f(*We*)^22^, we expect the step between both values of reduced time to occur at a critical Weber number larger than unity. We plot in Fig. 4 our data at intermediate and high impact velocities, for different drop and wire radii. The contact time normalized by its maximum value *τ*_o_ is plotted as a function of the Weber number *We*. Data collapse on a main step-shaped curve, and the step between the two regimes of reduced time is localized at *We*=7.5±1.5, and indeed independent of *b*.

**Figure 5:**
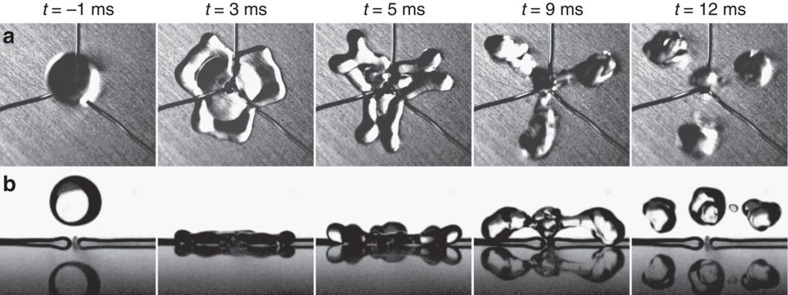
Bouncing of a water drop on a Y-pattern. Top (**a**) and side (**b**) views of a water drop (*R*=1.45 mm) impacting at *V*=0.9 m s^−1^ the symmetry centre of three wires of radius *b*=100 μm making an angle of 120° with one another. Water during impact makes six lobes that quickly merge in three symmetric subunits. The drop takes off at *t*=*τ*=9.0±0.2 ms, close to the time *τ*_o_/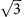
*=*8.7±0.4 ms expected from the scaling argument.

Hence the contact time of water drops bouncing symmetrically on a macrotexture is found to display discrete values depending on the impact velocity. We interpreted this observation from the number of quasi-independent subunits during impact, varying between 4, 2 and 1 in this study, hence providing contact times *τ*_o_/2, *τ*_o_/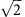 or *τ*_o_. This proposition has interesting consequences. First, if we consider not only symmetric shocks, but also fast off-centred impacts, distributions of mass on both sides of the wire become asymmetric. According to our arguments, the contact time should shift from *τ*_o_/2 to *τ*_o_, as the impact centre moves from the wire axis to the distance *R*_max_. Bird *et al.* reported such variations of *τ* as off-centring the impact^1^, and we display in Supplementary Fig. 2 a figure showing the continuous increase of *τ* between *τ*_o_/2 and *τ*_o_ as the distance between impact and wire is varied between 0 and 3 mm, for *R*=1.3 mm and *V*=1 m s^−1^. Supplementary Movie 6 shows an example of such an off-centred impact. Second, other patterns for the macrotexture will produce other distributions of mass, and thus other contact times. We tested a Y-pattern made of three wires starting from a unique centre and making an angle of 120° with one another. A drop impacting the symmetry centre of this device produces three independent subunits (Fig. 5; Supplementary Movies 7 and 8), so that we expect a contact time *τ*_o_/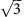. For *R*=1.3 mm, we observed this regime in a large interval of velocities (between 0.4 and 1.2 m s^−1^), with a constant contact time equal to 7.7±0.3 ms, indeed very close to *τ*_o_/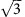
≈7.5±0.4 ms (see Supplementary Fig. 3 for the corresponding curves). The drop in Fig. 5 is larger, but contact time is similarly observed to be reduced by a factor 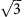.

Impacts at larger velocity on the same device lead to an even more reduced contact time (*τ*=6.4±0.2 ms), smaller than the minimum time observed with a simple wire, yet larger than *τ*_o_/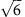
*=*5.3±0.3 ms expected for a drop divided into six independent lobes. Such a regime of high fragmentation is only attained at a large velocity (*V*>1.4 m s^−1^) for which lobes can detach from the rest of the drop, whose volume becomes ill-defined (Supplementary Movie 9). Hence we cannot predict accurately the contact time in this regime. More generally, many details of the reshaping of an impacting liquid by a given set of defects and the way it affects the contact time remain to be explored. A stimulating question concerns the design that would yield the absolute minimum of *τ*. On a repellent material, impacting water spends a few milliseconds close to the material, and it can be seen as an ultimate quest for repellency to reduce further and minimize this time.

## Methods

### Macrotextured substrate

Textures are made of nickel wires, of radius *b* varied between 25 and 250 μm. The wires are deposited on a polished aluminium surface (of 10 cm × 10 cm), and they are attached to weights on each side of the surface, which maintains them stretched and pressed against aluminium. Both surface and wire are treated with a commercial spray (Ultra Ever Dry, from Ultra Tech International), consisting of nanoparticles of ∼20 nm in size dispersed in acetone. When sprayed on the macrotextured surface, the solvent evaporates and leaves micrometric layers of nanoparticles that coat both the substrate and the wire. After spraying, we wait 30 min to ensure complete evaporation. The Y-pattern is made of three nickel wires of radius *b*=100 μm. They are held together in a hole of 400 μm drilled in the aluminium substrate, and again drawn tight by weights.

### Experimental procedure

Drops of deionized water are formed from five calibrated needles (Fisher Scientific), which enables a variation of the radius *R* of the drops between 1 and 2.3 mm. *R* is determined by measuring the weight of 10 drops on a precision balance. For each needle, the drop size is repeatable with <10%. By adjusting the height from which droplets fall, the impact velocity *V* can be varied between 20 and 150 cm s^−1^. Impacts are filmed from top or side views (or both when necessary) using Phantom V9 and Optronis CR600 cameras. Films are shot at 10,000 fps. From each movie, *V* is determined from the successive positions of the centre of mass of the droplets just before the impact, using the reslice tool of ImageJ software. Impact centring is adjusted using two manual stages and it is determined from top views. Contact time is defined between the moment when the south pole of the drop touches the surface and when the last fragment of liquid leaves the substrate.

## Additional information

**How to cite this article:** Gauthier, A. *et al.* Water impacting on superhydrophobic macrotextures. *Nat. Commun.* 6:8001 doi: 10.1038/ncomms9001 (2015).

## Supplementary Material

Supplementary Information Supplementary Figures 1-3

Supplementary Movie 1 The movie is a top view of an impact at V = 1.0 m s^−1^ of a water drop of radius R = 1.3 mm on a superhydrophobic wire of radius b = 100 μm maintained on a flat superhydrophobic surface. This movie corresponds to Figures 2a and 2b and it is slowed down by a factor of 500.

Supplementary Movie 2 This movie shows the same experiment as Supplementary Movie 1 (impact at V = 1.0 m s^−1^ of a water drop on a superhydrophobic wire of radius b = 100 μm) from the side and it is slowed down by a factor of 500.

Supplementary Movie 3 The movies shows a top view of the impact at V = 0.5 m s^−1^ of a water drop (R = 1.3 mm) on a superhydrophobic wire of radius b = 100 μm drawn tight on a flat repellent surface. This movie, which corresponds to Figures 2c and 2d, is slowed down by a factor of 500.

Supplementary Movie 4 This movie is the same experiment as Supplementary Movie 3 seen from the side (impact at V = 0.5 m s^−1^ of a water drop (R = 1.3 mm) on a superhydrophobic wire of radius b = 100 μm) and it is slowed down by a factor of 500.

Supplementary Movie 5 The movie is a top view of the impact at V = 0.3 m s^−1^ of a water drop of radius R = 1.3 mm on a superhydrophobic wire of radius b = 100 μm maintained on a flat superhydrophobic surface, an experiment for which the measured contact time is to, the time measured without texture. The movie is slowed down by a factor of 500.

Supplementary Movie 6 The movie shows an example of an off-centered impact at V = 1.1 m s^−1^ for a water drop of radius R = 1.3 mm. The distance x between the wire axis and the drop center is 1.2 mm and this top view shows a clear asymmetric distribution of mass on each side of the wire. The movie is slowed down by a factor of 230.

Supplementary Movie 7 The movie is a top view of a water drop (R = 1.45 mm) impacting at V = 0.9 m s^−1^ a Y-pattern made of three repellent wires of radius b = 100 μm forming an angle of 120° with one another. This movie corresponds to Figure 5 and it is slowed down by a factor of 500.

Supplementary Movie 8 This movie shows the same experiment as Supplementary Movie 7 seen from the side and it is slowed down by a factor of 500. (Impact of a water drop (R = 1.45 mm) impacting at V = 0.9 m s-1 a Y-pattern made of three superhydrophobic wires of radius b = 100 μm forming an angle of 120° with one another.)

Supplementary Movie 9 The movie shows a top view at a higher impact velocity (V = 1.6 m s^−1^). It is observed that the six lobes obtained on a Y-pattern detach rapidly (because of inertia), so that the bouncing mass becomes ill-defined.
