# Supplementary material for: Water impacting on superhydrophobic macrotextures
Source: Nat Commun. 2015 Aug 11;6:8001. doi: 10.1038/ncomms9001 (PMC4918367; doi:10.1038/ncomms9001)
Supplement: Supplementary Information — Supplementary Figures 1-3 [file ncomms9001-s1.pdf]

Supplementary Figures

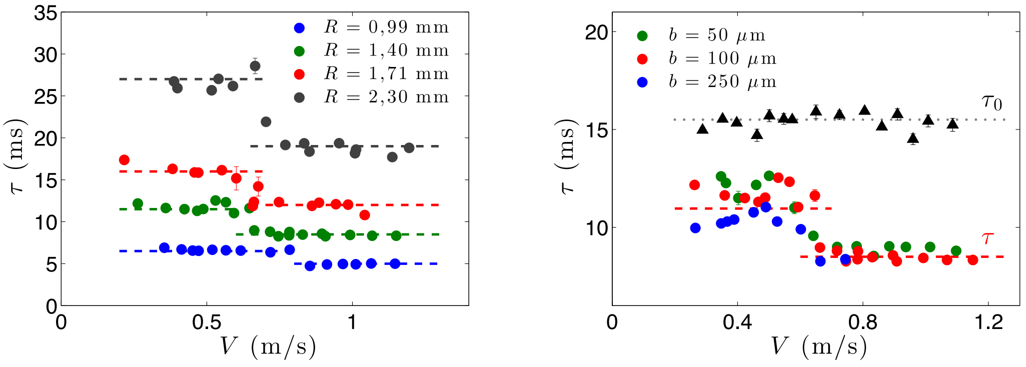

(a) **(b)**

Supplementary Figure 1. Contact time as a function of impact velocity for different drop and wire radii. a. Contact time τ on a surface textured by a wire of radius b = 100 µm as a function of impact velocity V, for various drop radii. Dashed lines are fits for τ. **b.** Contact times τ_0 on a regular superhydrophobic surface (black triangles) and τ on a textured surface, as a function of the impact velocity V for wires of radius b varying between 50 µm and 250 µm. The drop radius is constant and equal to R = 1.4 mm. Dotted and dashed lines are fits for τ_0 and τ. These raw data were used to build Figures 3b, 3c and 4 in the accompanying paper.

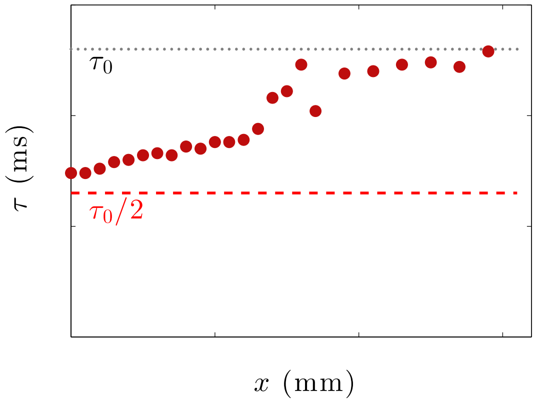

Supplementary Figure 2. Contact time for off-centered impacts. Contact time τ of water drops (radius R = 1.3 mm) bouncing after an impact at V = 1.1 m/s on a superhydrophobic material with a wire (b = 100 µm) of same repellency as a function of x, the distance between the wire axis and the drop center. Dotted and dashed lines show τ_0, the contact time measured without texture, and $\tau_0/2$, the contact time expected for a centered impact at such a velocity. From the arguments developed in the accompanying paper, we expect τ to scale as $(m/\gamma)^{1/2}$, where m denotes the volume of the largest fragment and γ the surface tension.

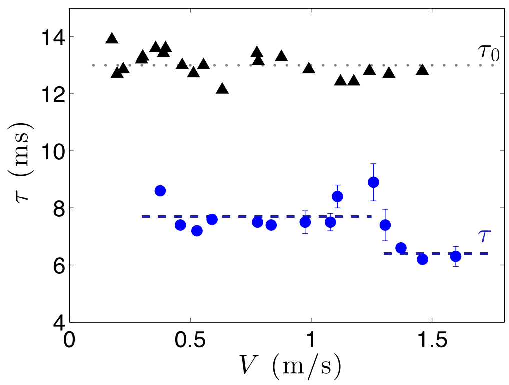

Supplementary Figure 3. Contact time for a drop bouncing on a Y-pattern. Contact time of water drops (radius R = 1.3 mm) impacting on a regular superhydrophobic material (time τ_0, black triangles), and on a surface textured by three wires (b = 100 µm) with a Y-pattern (time τ, blue circles) such as shown in Figure 5 of the accompanying paper. τ_0 and τ are plotted as a function of the impact velocity V; dotted and dashed lines are fits for the contact time in the two cases. At intermediate velocities, we have $\tau \approx 7.7 \pm 0.3$ ms, a value close to the expected value $\tau_0/3^{1/2} \approx 7.5 \pm 0.3$ ms. Above a threshold velocity, the contact time drops to $\tau \approx 6.4 \pm 0.2$ ms, which is smaller than any contact time reported in the accompanying paper, yet larger than $\tau_0/6^{1/2} \approx 5.3 \pm 0.2$ ms, the time expected if six independent lobes were bouncing independently.
